# Non-coding RNAs as key regulators in hepatitis B virus-related hepatocellular carcinoma

**DOI:** 10.3389/fimmu.2025.1602252

**Published:** 2025-06-23

**Authors:** Prasanna Srinivasan Ramalingam, Liming Zhang, Md Sadique Hussain, Gyas Khan, Wedad Mawkili, Ali Hanbashi, Gaurav Gupta, Purushothaman Balakrishnan, Sivakumar Arumugam

**Affiliations:** ^1^ Protein Engineering Lab, School of Biosciences and Technology, Vellore Institute of Technology, Vellore, Tamil Nadu, India; ^2^ School of Basic Medical Sciences, Tsinghua University, Beijing, China; ^3^ Uttaranchal Institute of Pharmaceutical Sciences, Uttaranchal University, Dehradun, Uttarakhand, India; ^4^ School of Pharmaceutical Sciences, Lovely Professional University, Phagwara, Punjab, India; ^5^ Department of Pharmacology and Toxicology, College of Pharmacy, Jazan University, Jazan, Saudi Arabia; ^6^ Centre for Research Impact and Outcome-Chitkara College of Pharmacy, Chitkara University, Rajpura, Punjab, India; ^7^ Centre of Medical and Bio-allied Health Sciences Research, Ajman University, Ajman, United Arab Emirates; ^8^ Department of Biomaterials, Saveetha Dental College and Hospitals, SIMATS, Saveetha University, Chennai, India

**Keywords:** biomarkers, hepatitis B virus, hepatocellular carcinoma, non-coding RNAs, therapeutic targets

## Abstract

Hepatitis B virus (HBV)-related hepatocellular carcinoma (HCC) remains a significant global health challenge due to its high prevalence and poor prognosis. Recent advances have revealed that non-coding RNAs (ncRNAs), including microRNAs, long ncRNAs, circular RNAs, and small nucleolar RNAs, play critical regulatory roles in HBV-induced oncogenesis. These ncRNAs modulate various cancer hallmarks and contribute to HCC progression. Notably, their stability, detectability in bodily fluids, and disease-specific expression patterns render these ncRNAs as highly promising diagnostic and prognostic biomarkers for HBV-HCC. Herein, we review the types and mechanisms of HBV-related ncRNAs, emphasizing their dual roles as oncogenes and tumor suppressors. Furthermore, we discuss their applicability as diagnostic markers and therapeutic targets and review recent directions in ncRNA-based approaches that aim to enhance patient treatment. Concerning these aspects, the present review aimed to provide an understanding of the complexity of ncRNAs in HBV-related HCC with the hope of directing future research and developments towards effective control of this complex malignancy known as HCC.

## 1 Introduction

Hepatitis B virus (HBV) is a widespread pathogen and a principal agent of hepatocellular carcinoma (HCC), a significant subset of liver tumors. Globally, HCC is the fifth most common cancer and the third leading cause of cancer-related mortality. Chronic HBV infection impacts more than 296 million individuals worldwide, placing many at heightened risk for severe liver conditions such as cirrhosis and HCC (1, 2)​. HBV is particularly problematic in regions like Asia and Africa, where its prevalence and associated HCC cases are alarmingly high​ (3). While vaccination programs have reduced HBV incidence, the burden of HBV-related HCC remains significant, contributing to around 80% of HCC cases globally (4)​. The chronic inflammatory processes induced by persistent HBV infection, along with viral factors such as the HBV-encoded X protein (HBx), contribute to tumorigenesis even in the absence of cirrhosis (5). Non-coding RNAs (ncRNAs), encompassing categories such as long ncRNAs (lncRNAs), microRNAs (miRNAs), circular RNAs (circRNAs), and small nucleolar RNAs (snoRNAs), play essential roles in regulating gene expression and have become a central topic of interest for their contribution to cancer research (6). These ncRNAs, while not coding for proteins, influence various oncogenic processes, including those driven by HBV. Given the HBV-related HCC, ncRNAs regulate the TME, inflammation, and metabolism influencing cancer development. For example, HBx is also reported to change the expression of ncRNA, thus participating in immune regulation and uncontrolled cell proliferation (5)​. New studies show that while some ncRNAs promote tumorigenesis, others act to inhibit cancer growth.

**Figure 1:**
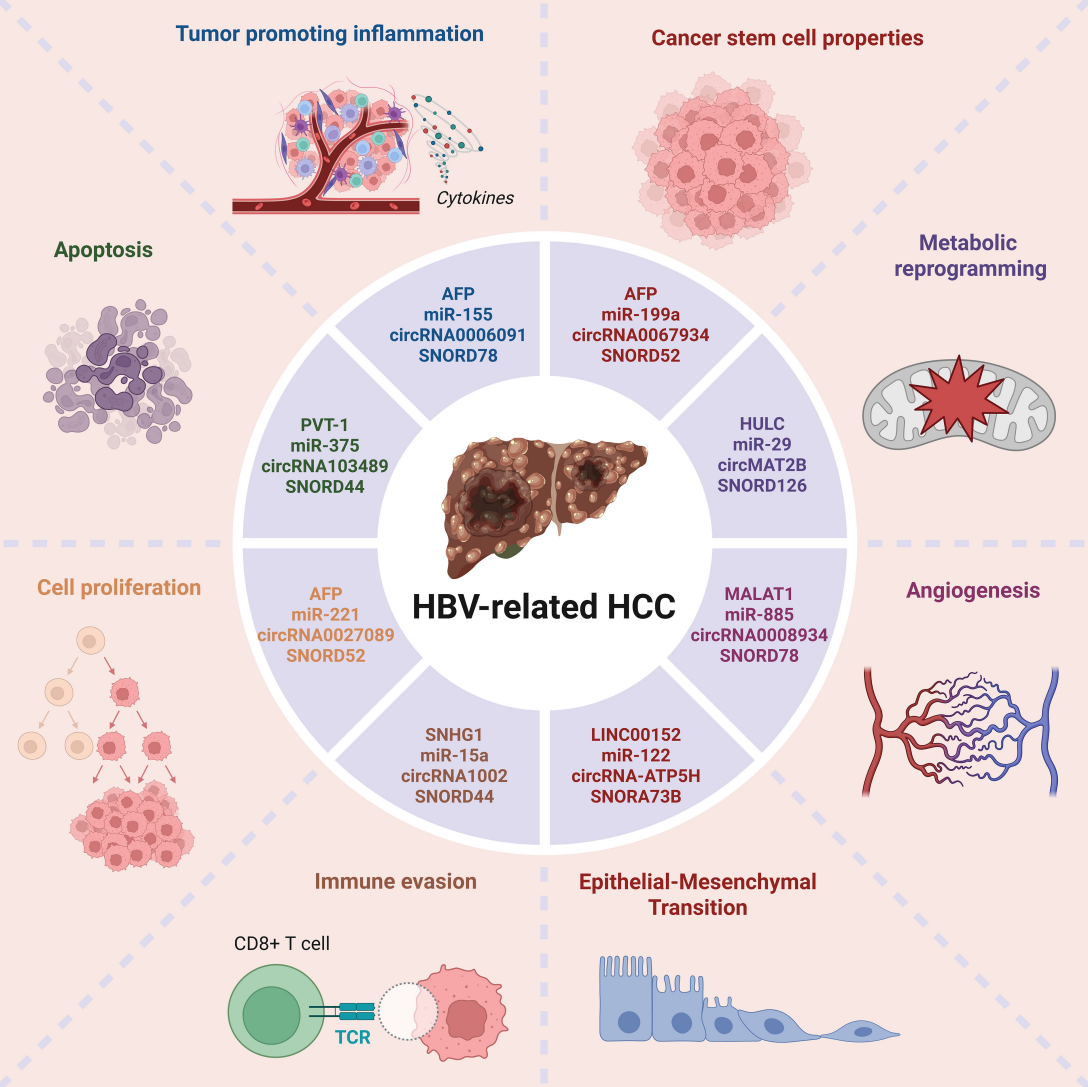
ncRNAs in HBV-related HCC involved in cancer progression. ncRNAs such as the lncRNAs, miRNAs, and circRNAs involved in various mechanism including apoptosis, proliferation, immune evasion, tumor promoting inflammation, etc.

NcRNAs play an essential role in HBV-induced HCC development and progression, through precisely modulating the expression of their target genes and associated cellular processes. In various classes, ncRNAs regulate major processes including cell division/programmed cell death, immune response, and metastatic processes as shown in **Figure 1**. For instance, miRNAs such as miR-155 and miR-135a are involved in tumor formation or inhibition following the regulation of certain mRNAs and commanding pathways, for instance, MAPK and Wnt/β-catenin (7, 8). Overall, LncRNAs including DLEU2 and HOTAIR are implicated in chromatin regulation, transcription regulation, and cell cycle constraining or enhancing tumor formation (9). CircRNAs participate in this process by binding to miRNAs thus infringing on their regulation (10). Altogether, these ncRNAs modulate the TME, help escape immune regulations, and contribute to metabolic alterations, making them ideal biomarkers and promising targets in HBV-related HCC.

Given the high incidence and mortality of HBV-HCC, especially in regions, where there is poor access to early diagnostics and personalized therapy, the urgent need arises for new reliable molecular regulators, which can have diagnostic and therapeutic functions as well. Because of tissue-specific exposition, regulatory capabilities, and distribution in body fluids ncRNAs have remarkable potential in this field. This article aims to explore all the multiple roles of HBV-associated ncRNAs as far as the disease development of HCC is concerned. Through the elucidation of two key regulatory mechanisms and their effects on tumor advancement, we hope to explain how ncRNAs can serve as future therapeutic targets for the treatment of HBV-induced HCC. This review will identify major ncRNAs implicated in HBV-related oncogenesis, and describe their biological roles and possible treatment strategies. We further place emphasis on recent discoveries, point to translational problems, and attempt future perspectives, thus presenting a unified resource both to researchers and clinicians of liver cancer research.

## 2 Types of HBV-related ncRNAs

### 2.1 MiRNAs

miRNA is a group of small endogenous ncRNA of about 21–25 nucleotides in length involved in the regulation of gene expression at the post-transcriptional level. Its action is mediated through binding to complementary sequences on target mRNAs, thereby resulting in degradation of the message or inhibition of its translation (11, 12). When it comes to HBV-associated HCC, global alternation in the expression of miRNA plays an important role in disease progression (13). Some miRNAs are upregulated, contributing to oncogenic processes, while others are downregulated, often functioning as tumor suppressors (14). The biogenesis of miRNAs involves multiple steps. miRNAs are first synthesized as primary transcripts, known as pri-miRNAs, which undergo nuclear processing by the Drosha enzyme to generate precursor miRNAs (pre-miRNAs). These precursors are subsequently exported to the cytoplasm, where they are cleaved by the Dicer enzyme to produce mature miRNAs. The mature miRNAs are then assembled into the RNA-induced silencing complex (RISC), enabling them to bind to target mRNAs and modulate gene expression (15, 16).

In HBV-related HCC, several miRNAs are upregulated. MiR-21, regulated by HBx, represents among the critical consistently upregulated miRNAs that acts as oncogenic and targets PDCD4 & PTEN in HBV-related HCC (17). Its elevated expression is strongly linked to increased tumor cell growth and survival. In addition, miR-221 exerts oncogenic & miR-222 exerts both oncogenic & tumor suppressor roles and are also commonly upregulated by HBx and by targeting CXCL4/12 and transferrin receptor protein 1 (TFRC) respectively, and play key roles in facilitating cell cycle advancement while suppressing apoptosis, thus supporting cancer cell growth (18, 19). Likewise, the oncogenic miR-17–92 cluster, including miR-18a, miR-19a/b, and miR-92a, are also upregulated and targets ERα in HBV-HCC (20). Also, they contributes to proliferation, angiogenesis, and immune evasion. Additionally, hypoxia-related miRNA miR-210-3p is also up-regulated and exerts an oncogenic role by targeting HIF-1α & FGF1 in HBV-related HCC. Increased expression of this protein aids tumors to survive in conditions where oxygen is scarce, therefore encouraging the formation of new tumors (21, 22). Lastly, oncogenic miR-96 that targets Glycoprotein M6a (GPM6A) is also highly expressed in HCC and works positively in promoting proliferation and growth by negatively targeting tumor suppressor genes (23, 24).

Conversely, several miRNAs with tumor-suppressive functions are downregulated in HBV-associated HCC. Firstly, miR-122 is a liver miRNA that is crucial for HBV infection, and it is downregulated by IL-6 and TNF-α, and its absence is associated with the promotion of tumor development, motility, and invasion (25). These HCC-specific metabolic changes affect tumor aggressiveness through the downregulation of miR-122. For instance, miR-122 downregulation is associated with the changes in anaerobic glycolysis by targeting pyruvate kinase M2 (PKM2), and amino acid metabolism by targeting Solute carrier family 7 member 1 (SLC7A1) respectively (26). Likewise, tumor-suppressive miR-199a/b inhibits ROCK1/MLC and PI3K/Akt pathways via targeting the Rho-associated coiled-coil kinase 1 (ROCK1) in HBV-HCC, and its low expression is also associated with poor overall survival (27). To note, miR-125b acts as tumor-suppressive and inhibits angiogenesis by targeting VEGFA, and induces cell-cycle arrest by directly targeting cyclin D2/E2 & IL-6-stat3 signaling pathway in HCC (28, 29). Another tumor-suppressive miR-101 is down-regulated by HBx was reported to induce aberrant DNA methylation in HCC by targeting DNA methyltransferase 3A (DNMT3A) (30). In HBV-HCC, MiR-29 was reported to modulates the apoptosis & cancer stem cell properties by inhibiting BCL-2 expression levels (31). Furthermore, miR-148a and miR-152 were also reported to be downregulated by HBx, and their expression was associated with AKT/ERK/FOXO4/ATF5 pathway repression via HPIP inhibition and repressed RIZ1 expression via DNMT1 in HCC (32, 33). Moreover, miR-30a-5p is another downregulated miRNA in HBV-related HCC, that influences the dynamics of the EMT transition via targeting Snail family transcriptional repressor 1 (SNAIL1) (34).

### 2.2 LncRNAs

LncRNAs represent a diverse class of RNA molecules longer than 200 nucleotides, characterized by the absence of protein-coding capability. Transcribed by RNA polymerase II, these molecules undergo typical post-transcriptional modifications such as 5’ capping and 3’ polyadenylation (35). Based on the location of the source, lncRNAs are known to form several categories, these include; intergenic lncRNAs depending on source regions in between genes and antisense lncRNAs emanating from the opposite strand to protein-coding genes (36, 37). Its functional modes vary greatly and include interactions with DNA, RNA, or proteins. As far it is known, lncRNAs can modulate genes in different ways if not directly involving genetic codes in the sense of DNA, RNA, and peptides, as follows, chromatin modification, transcriptional regulation, and post-transcriptional regulation (38). Also, they can be used as molecular support for oligonucleotides, protection for nucleic acids, signal for molecular traffic, and co-activators or co-repressors in the regulation of such processes as gene silencing, splicing, and transcription activation (39). LncRNAs are involved in a wide range of cellular processes such as cell development differentiation, and disease progression. With regards to HCC especially in the context of chronic HBV, a great many lncRNAs have been identified that are up or down-regulated and have been implicated in the initiation and progression of liver cancer (40). Thus, the lncRNA expression profiles of HBV-associated HCC have attracted significant attention in addressing this frequently invasive cancer.

The dysregulation of lncRNAs in HBV-related HCC impacts various hallmarks such as cellular proliferation, invasion, metastasis, angiogenesis, and immune evasion by targeting their mRNA targets via miRNA sponging. HULC is one of the most investigated lncRNAs in the context of HBV-related HCC, which was observed to be significantly upregulated in hepatic cancer tissues (41). It is also evidenced that HULC positively regulates cellular growth and modulates various signaling pathways such as MAPK, NF-κB, PI3K/AKT, Wnt/β-catenin, and Hippo by sponging the miRNAs miR-101, miR-200a, miR-372, miR-34a, miR-186 respectively and involves in HCC progression (42). Another important lncRNA associated with HBV-related HCC is High Expression in Hepatocellular Carcinoma (HEIH), which was reported to promote liver cancer and regulates the cell cycle via miR-129→ EZH2 in HepG2.2.7.1 cells (43). Additionally, HEIH was also reported to be related to poor prognosis in cases of HBV-related HCC patients, its role becomes important in defining the aggressive nature of the disease. Similarly, upregulated Urothelial Cancer Associated 1 (UCA1) was also observed to be increased after HBx in HBV-associated HCC, and promotes tumor growth and metastasis via recruiting EZH2 and repressing p27Kip1/CDK2 signaling in patients with chronic HBV (44). In addition, overexpressed DLEU2 was also reported to promote tumor growth and progression via targeting the EZH2 in HBV-related HCC (9).

Likewise, HOX Transcript Antisense RNA (HOTAIR), which was originally detected in other types of cancer, is particularly upregulated in HBV-associated HCC owing to the activation of HBx via two transcriptional repressive complexes such as Polycomb Repressive Complex 2 (PRC2) & lysine-specific demethylase 1/corepressor/RE1-silencing transcription factor (LSD1/CoREST/REST) complex respectively. This lncRNA is well correlated with enhanced malignant phenotype including tumor metastasis (45). This molecule is highly expressed in advanced disease and associated with poor prognosis among patients. Apart from these identified lncRNAs, prior investigations revealed several more lncRNAs linked with HBV-induced HCC and mechanistically promotes HCC progression. lncRNA DBH-AS1 expression was positively correlated with Hepatitis B surface antigen (HBsAg) and is conducive to tumor development by regulating cellular proliferation and metastasis (46). Likewise, the upregulated LINC00152 is associated with HCC progression via involving in the regulation of the proliferation and metastasis (47). Both of these lncRNAs have been mentioned in previous studies highlighting their possible function in aggravating the effects of chronic HBV infection on hepatic tissue. Additionally, LncRNA-ATB (Activated by Transforming Growth Factor-beta) upregulation was also reported to promote HCC progression by regulating metastasis (48). Furthermore, the Metastasis Associated Lung Adenocarcinoma Transcript 1 (MALAT1) was also often over-expressed and directly associated with associated HBV-associated HCC progression (49). Collectively, it was observed that these lncRNAs promoted HBV-related HCC by potentially regulating the proliferation & metastasis.

However, some lncRNAs are expressed at lower levels in HBV-associated HCC and have a tumor suppressor effect; their downregulation plays an oncogenic role. MEG3, short for Maternally Expressed Gene 3, is a relatively well-known tumor-suppressive lncRNA, whose expression is often downregulated in HBV-HCC (50). MEG3 has tumor-suppressive properties since it regulates the proliferation of the cells and induces apoptosis and low levels of MEG3 mRNA correlate with increased tumor growth rate and poor survival. The downregulation of MEG3 has been reported in several works presented in this review and therefore, plays a critical role in normal liver function if lost in cancer. Also, GAS5, reduced in HBx affects cell cycle arrest and apoptosis, and its downregulation aids in cancer cell survival (51).

### 2.3 CircRNAs

circRNAs are a distinct class of ncRNAs characterized by their circular structure derived from precursors with a covalent bond at the terminal. CircRNAs are formed through back-splicing which is the joining of the 3’ and the 5’ end of the pre-mRNA making a circle. CircRNAs can be from exons, introns, or both across their backspliced arcs. The circular structure provides them more stability and protection from exosomes which act through exonuclease. CircRNAs mainly exert their effects through the molecular function of “miRNA sponges” that prevent the miRNA from exerting its effect on the targeted mRNAs (52). In addition, circRNAs are capable of binding to RNA-binding proteins that affect their function and can be translated into small peptides in some cases. Recent research has underlined their causal and promoting roles in the development of HCC, especially in the context of chronic HBV. Differential expression of circRNAs which act as “sponges” for miRNAs, meaning that their activity can inhibit miRNAs from binding to their target mRNAs. Several circRNAs have been identified in HBV-related HCC that are involved in regulating oncogenic signaling and enhancing tumorigenesis (53).

Due to their compact co-circular conformation, circRNAs are protected from degradation and many circRNAs are reported to be upregulated in tumor tissue compared with healthy tissue. We now understand that in HBV-related HCC, these circRNAs are upregulated and play a role in cancer promotion. There are circRNAs such as Circ_0027089 are often upregulated in HBV-related liver tumors, and are associated with increased tumor proliferation (54). Another upregulated circRNA is Circ_100338; it also promotes metastasis in HBV-related HCC (55). circ_101764 was also upregulated in HBV-related HCC patients and its elevated levels were associated with poorer prognosis of the disease (56). Along with these, circ-ARL3 has been identified as markedly elevated in HBV-related HCC through m6A modification indirectly induced by HBx, and its increased expression is connected with the presence of liver cancer advancement (57). Similarly, circRNA_100338 is another circRNA, highly expressed in tumor tissues and linked to unfavorable outcomes in HBV-related HCC patients (58). Circ_0009910 and circ_0001649 are other examples of circRNAs found at higher levels in HBV-related HCC tissues, highlighting the broad scope of circRNAs that contribute to the disease’s progression (53).

Conversely, some circRNAs are downregulated in HBV-related HCC and are often linked to the loss of tumor-suppressive functions. For instance, circMTO1 is known to suppress tumor proliferation by sponging miR-9, while circZKSCAN1 functions to suppress cell migration and invasion (59, 60). Similarly, circTRIM33–12 is involved in modulating key pathways including TGF-β signaling, contributing to the suppression of HBV-related HCC progression (61). The suppression of these circRNAs impairs their control over oncogenic miRNAs and key signaling pathways, leading to enhanced cancer cell proliferation, invasion, and metastasis in HBV-related HCC. Consequently, these circRNAs hold potential as diagnostic biomarkers or therapeutic targets to reinstate their tumor-suppressive roles.

### 2.4 SnoRNAs

SnoRNAs are a class of small RNA molecules, typically 60 to 300 nucleotides long, that play a central role in chemically modifying other RNAs, such as ribosomal RNA (rRNA), transfer RNA (tRNA), and small nuclear RNA (snRNA). They originate from the introns of host genes and accumulate in the nucleolus, where they direct the precise modification of rRNA through 2’-O-methylation or pseudouridylation (62). SnoRNAs are classified into two main groups: C/D box snoRNAs guide methylation processes, while H/ACA box snoRNAs are involved in directing pseudouridylation. SnoRNAs are crucial for the accurate assembly and functionality of ribosomes, and by extension, for protein synthesis. Although primarily associated with ribosomal biogenesis, Recent research suggests that snoRNAs may additionally contribute to gene expression regulation and are involved in disease mechanisms, including cancer (63). Recently, their roles in cancer biology, particularly in HBV-related HCC, have become more prominent. Several snoRNAs have been noted for their disrupted regulation in HBV-related HCC, playing crucial roles in either promoting or suppressing cancer development.

In HBV-related HCC, several snoRNAs are upregulated and contribute to tumor progression. Wang and colleagues observed that snoU2_19, SNORD78, and SNORD76 were overexpressed in HCC tissues infected with HBV. SnoU2_19 is linked to the activation of oncogenic signaling pathways (64). It has been identified as being involved in pathogenesis as well as the progression of HCC and is therefore regarded as a potential therapeutic target. Further, other studies have pointed out that SNORD76 was upregulated in hepatic cancer tissues with a connection to HBV (65). High levels are associated with higher proliferation of cancer cells and metastasis. ACA11 is another snoRNA that is up-regulated and has been confirmed to promote tumor development in HBV-related HCC by stimulation of cell proliferation and invasion (66). Another example of upregulated snoRNAs in HBV-related HCC is SNORD78, of which qPCR analysis revealed 47 HBV-related surgical samples of HCC had higher SNORD78 expression levels than in non-cancerous liver tissues. The overexpression of SNORD78 levels was tightly associated with a higher tumor stage and worse survival of HBV-related HCC patients. In addition, positive SNORD78 expression was significantly associated with poor overall survival and shorter recurrence-free survival times (67). In addition, SNORD88B is upregulated in liver CSCs (CD13+CD133+ cells) and is involved in the HCC tissues with HBV infection (68). It promotes the self-renewal capacity of hepatic CSCs and drives HCC tumor development through a non-canonical mechanism.

On the other hand, during the progression of HBV-related HCC, different snoRNAs perform unique regulatory roles. For example, SNORD31 is significantly underexpressed in HCC, and its reduced expression is closely associated with increased tumor size, a higher incidence of vascular embolism, extensive capsular invasion, and poor tumor differentiation (69). Meanwhile, SNORD17 plays a suppressive role in HCC progression, though its expression is repressed, contributing to the advancement of the disease (70). Research has identified that several snoRNAs, including SNORD113-8, SNORD113-5, and SNORD114-1, are downregulated in HBV-related HCC, where they function as tumor suppressors (64). However, the role of SNORD114–1 in HCC progression is still unknown; Nevertheless, it has been described as a tumor suppressor in leukemia due to the G0/G1 cell cycle arrest and cell proliferation reduction (68, 71).

## 3 Mechanisms by which ncRNAs promote HBV-related HCC progression

**Figure 2:**
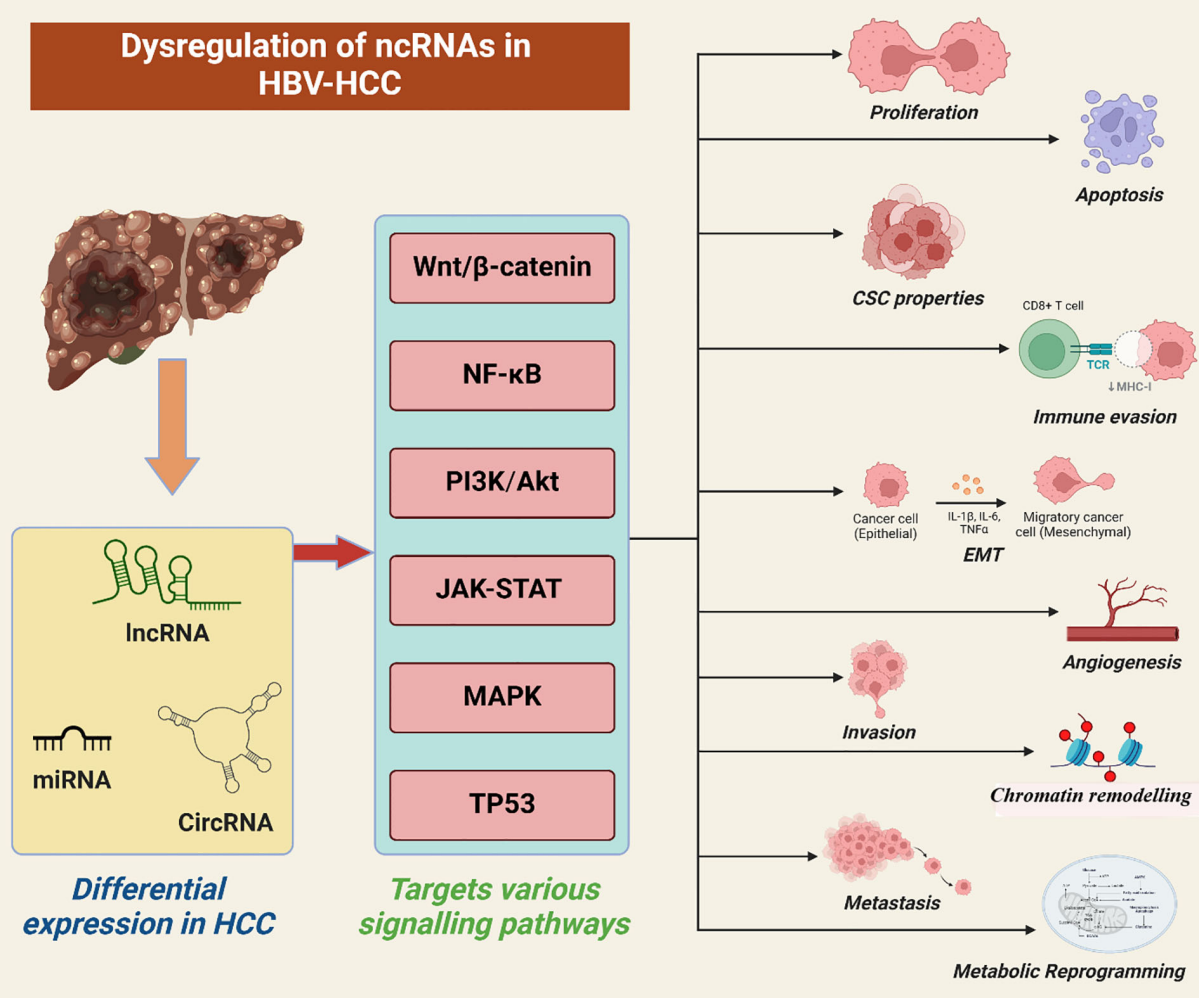
Aberrant expression of ncRNAs in HBV-related hepatocellular carcinoma has been observed. Different types of ncRNAs, including lncRNAs, miRNAs, and circRNAs, exhibit altered levels in HCC and interact with various proteins, influencing multiple signaling pathways such as MAPK, PI3K/Akt, NF-κB, Wnt/β-catenin, JAK-STAT, and TP53. Also, via regulating these pathways, the ncRNAs mechanistically regulates multiple mechanisms in HBV associated HCC including proliferation, invasion, metastasis, apoptosis, chromatin remodeling, immune evasion, EMT transition, cancer stem cell properties, angiogenesis and metabolic reprogramming of energy and glucose metabolism respectively.

The current studies have also demonstrated that ncRNAs are vital in promoting the development of HBV-related HCC by controlling basic functions such as cell division, apoptosis, metastasis, immune escape, and self-renewal. **Figure 2** shows the dysregulation of ncRNAs in HBV-related HCC.

### 3.1 Promotion of uncontrolled cell proliferation

In HBV-related HCC, loss of control over some molecules such as cyclins and cyclin-dependent kinases (CDKs) may take a break off the cell cycle hence allowing the cancerous cells to proliferate continuously. miR-187-5p is downregulated by HBx in HBV-related HCC and inhibits tumor cell proliferation by targeting the E2F1/FoxP3 axis. E2F1 serves an essential function in promoting the transcription of diverse cyclins and CDKs, especially those involved in the transition from the G1 to the S phase of the cell cycle (72). Normally, miR-187-5p acts as a tumor suppressor by blocking cell cycle progression, and when miR-187-5p is absent due to HBx inhibition, E2F1 expression increases, facilitating the G1 to S phase transition and driving unchecked cell proliferation.

Furthermore, circRNA hsa_circ_0005218 controls cell cycle progression by sponging miR-31-5p, which normally inhibits CDK1 expression (73). In HBV-positive HCC, hsa_circ_0005218 is upregulated, leading to enhanced CDK1 expression, promoting the G1/S phase transition and driving rapid tumor cell proliferation. Patients with elevated circ_0005218 expression exhibited poorer overall survival (OS) and disease-free survival (DFS). Furthermore, LINC02532 promotes the proliferation of HBV-related HCC by targeting miR-455-3p, which suppresses CHEK2, a key DNA damage checkpoint kinase (74). High CHEK2 expression or phosphorylated form may lead to chromosome lag and might have a role in the promotion of HCC by affecting normal mitotic progression (75). These lagging chromosomes are supervised by a spindle assembly checkpoint that controls the activity of Cyclin B/CDK 1. Evaluation of LINC02532 overexpression in HBV-infected cells revealed that it enhances CHEK2 mRNA expression and progression of the cell cycle regulating proliferation. Last but not least the upregulated lncRNA MAPKAPK5_AS1 in HBV-related HCC tissues promotes the transcription of CDK4/6 and S-phase kinase-associated protein 2 (Skp2) by interfering with extra-cellular signal-regulated kinase (ERK) pathway mediated degradation of c-Myc protein (76). This stabilization enhances c-Myc-dependent cell cycle progression through the G1 and S phases.

For instance, circ_0027089 promotes HCC progression by sponging miR-136-5p, which normally inhibits NACC1, a key oncogenic factor (54). By preventing miR-136-5p from inhibiting NACC1, circ_0027089 activates the Wnt/β-catenin and PI3K/AKT pathways, which are integral to tumor cell growth. HBV infection upregulates circ_0027089, contributing to the rapid growth of HCC cells, and animal model studies confirm that circ_0027089 knockdown leads to reduced tumor growth. Additionally, miR-128-3p is downregulated by HBx in HBV-positive HCC, allowing for the stimulation of the JNK pathway via SPG21, and is associated with worse overall survival (77). This activation promotes tumor cell growth by enhancing calcium influx through TRPM7. Additionally, miR-135a targets HOXA10, a key modulator of the MAPK/ERK pathway, and its downregulation in HBV-positive HCC leads to increased HOXA10 expression, which drives cell proliferation through the MAPK pathway (7). This contributes to the increased growth and survival of HCC cells. Likewise, HBV promotes the proliferation of hepatic cancer cells by regulating the hsa_circ_0000847/miR-135a axis (78). Through this pathway, HBV promotes the phosphorylation of p38, ERK, and JNK, further activating the MAPK signaling pathway. hsa_circ_0000847 binds to miR-135a, controlling cell growth.

SNHG18 functions as a negative regulator of tumor advancement, and its downregulation in HBV-related HCC removes the inhibition of key tumor suppressor pathways, leading to increased proliferation (79). Additionally, SNHG18 mediates the effects of oleanolic acid, a natural compound that has shown antitumor effects by upregulating SNHG18 expression. Furthermore, hsa_circ_0001964 promotes tumor growth in HBV-related HCC by inhibiting the PI3K/AKT signaling cascade and its downstream effector, such as c-Jun, c-Myc, and CCND, which is involved in cell cycle arrest (80). Its upregulation in HCC cells allows for unchecked proliferation, driving tumor progression. Moreover, OIP5-AS1 exerts tumor-suppressive effects through the regulation of the glycolysis pathway through HKDC1 (81). In HBV-infected cells, OIP5-AS1 downregulation resulted in upregulation of HKDC1, which promotes glycolysis and accelerates cell cycle progression, thereby driving tumor growth and also it showed a reduction in expression in HBV-positive HCC patients.

### 3.2 Suppression of apoptosis and cell death

In HBV-related HCC, ncRNAs inhibit apoptosis through many different mechanisms, including the degradation of molecules that are important for apoptosis and regulation of the mitochondrial pathway of apoptosis. For instance, miR-96-5p negatively regulated the proapoptotic tumor suppressor gene GPM6A. In the HBV-positive HCC sample, it was also found that miR-96-5p is upregulated and leads to the downregulation of GPM6A hence inhibiting the apoptotic process. This supports the survival of the tumor cells and their resistance to apoptosis (24). Another study provides evidence that down-regulation of CircCCNB1, a miR-106b-5p binder, inhibited GPM6A level and promoted liver cancer cell apoptosis by increasing DYNC1I1 level (82). Additionally, miR-642a-5p controls apoptosis through the downregulation of DDIT4 protein which is a known apoptosis pathway controller (83). LINC00238 is significantly reduced in HBV-related HCC and functions as a tumor inhibitor by promoting apoptosis (84). HBV infection inhibits LINC00238 expression, and overexpression of LINC00238 has been shown to suppress HBV replication. Mechanistically, LINC00238 inhibits the expression of transmembrane protein 106C (TMEM106C), which contributes to suppressing apoptosis pathways, including caspase-7 and programmed cell death 4 (PDCD4) (85). CircMRPS35 exhibits elevated expression in HBV-related HCC and accelerates tumor advancement by hindering apoptosis (86). Mechanistically, circMRPS35 functions as a sponge for miR-148a, leading to the upregulation of Syntaxin 3 (STX3), which regulates the ubiquitination and degradation of PTEN. Similarly, circDDX17 is hindered in HBV-related HCC and acts as a tumor inhibitor by regulating the miR-21-5p/PTEN axis (87).

The mitochondrial apoptosis pathway is mainly regulated by proteins of the Bcl-2 family, which encompass both pro-apoptotic members like BAX and BAK, and anti-apoptotic members such as Bcl-2. They regulate the permeability of the mitochondrial membrane, which is a critical process in the release of cytochrome c, followed by caspase activation, causing apoptosis. Among them, MiR-3188 plays an important role in modulating apoptosis in HBV-infected cells (88). Forced upon by HBV-related HCC, miR-3188 was downregulated hence increasing the level of Bcl-2. In a normal state, the miR-3188 inhibits Bcl-2 so that it activates other pathways that lead to apoptosis and subsequent cell death. However, in the context of HBV infection, down-regulation of miR-3188 leads to up-regulation of Bcl-2, preserving the survival of HBV-infected reside, indicating that it is less sensitive to apoptosis-inducing treatments. Further, HBV_circ_1 enhances the HCC progression through suppression of mitophagy, a type of autophagy removing damaged mitochondria (89). HBV_circ_1 directly binds to PHB2 an intramitochondrial protein involved in mitophagy and inhibits its interaction with LC3, a protein that plays a crucial role in an autophagy pathway. HBV_circ_1 inhibits mitophagy and thereby suppressing apoptosis and promoting cell survival. HBV-related HCC has a positive association between evasion of apoptosis and tumor proliferation.

### 3.3 Modulation of tumor spread

NcRNAs are shown to inhibit HBV-related HCC cell migration, invasion, and metastasis via several pathways. These include control of the deposition of the extracellular matrix (ECM), encouragement of EMT, and the alteration of affinity placed on angiogenesis, all of which facilitate metastasis. Tumor spread is a crucial consideration in cancer progression cause cancer cells spread to different organs which are typically fatal to the patient. Firstly, miR-424-3p is reduced in HBV-related HCC, leading to the enhanced production of matrix metalloproteinases (MMPs) through reducing the interferon pathway via reducing the transactivation of STAT1/2 and IRF9 genes by SRF, resulting in ECM degradation (90). Similarly, circSFMBT2, significantly reduced by HBx, is linked to unfavorable outcomes and vascular infiltration in HCC (91). Elevated expression of circSFMBT2 suppresses metastasis in both laboratory and animal models, and circSFMBT2 promotes the sequestration of miR-665, which inhibits tissue inhibitors of metalloproteinases 3 (TIMP3). HBx reduces circSFMBT2 expression through interaction with DExH-box helicase 9 (DHX9), a protein that binds to introns flanking circRNA-forming regions.

Notably, circSMAD2, downregulated in HBV-related HCC serves as a suppressor of tumor growth by sponging miR-629, leading to increased PDCD4 expression which ultimately inhibits EMT (92–94). Inversely, circSETD2 is downregulated in HBV-related HCC, promoting cancer progression by enhancing EMT (95, 96). HBV suppresses circSETD2 expression, which in turn upregulates EMT markers like Vimentin and N-cadherin, facilitating tumor metastasis. Additionally, LINC00924 sponges miR-6755-5p is reduced in HBV-related HCC and inhibits EMT and tumor invasion (97) TSPEAR-AS1 is under-expressed, and sponges miR-1915-5p in HBV-related HCC, and inhibits metastasis & regulates EMT via NDRG2 (98). LINC00665 is elevated in HBV-related HCC that regulates angiogenesis by modulating the miR-126-5p/VEGFA axis and is linked to poor prognosis (99). HBV, through its surface antigen HBsAg, upregulates LINC00665 expression by triggering the NF-κB pathway (100). Additionally, HBV-miR-3 elevation was also reported to enhance angiogenesis by repressing the production of Factor Inhibiting Hypoxia-inducible factor 1 (FIH-1), which promotes angiogenesis via HIF-1α/VEGFA axis (101).

### 3.4 Modulation of immune evasion and tumor microenvironment

In HBV-related HCC, ncRNAs participate in the regulation of immune escape and tumor immune microenvironment through regulation of immune checkpoints, cytokines secretion, and immunosuppressive cell infiltration. To note, AC099850.3, a gene that is responsible for immune evasion, promotes HBV-associated HCC by CD276 overexpression, an immune checkpoint protein that inhibits immune recognition of tumor cells (102). Further, the lncRNA TUG1 has up-regulated expression in HBV-associated HCC and is involved in positive regulation of immune checkpoint (103). METTL3 promoted immunity-related protein expression, including PD-L1 and CD47, through m6A modification of the TUG1 gene to facilitate cancer immune evasion. TUG1 regulates PD-L1 and CD47 through absorbent of miR-141 and miR-340 and then restraining the activation of CD8+ T cells and the phagocytosis of macrophages. Regulating cytokine production is one of the mechanisms that are seen with ncRNAs in interaction with immune evasion and molding of the tumor environment. MEG3, a tumor suppressor lncRNA, is generally downregulated in HBV-associated HCC (104). Under normal circumstances, MEG3 regulates the secretion of pro-inflammatory cytokines such as IFN- γ and TNF-α in combination with miR-214. In the context of HBV infection, the weakened anti-cancer immune effect is due to decreased levels of IFN-γ and TNF-α as a consequence of the loss of MEG3. Furthermore, in HBV-related HCC, it can also participate in the regulation of immune response through the transformation of tumor-associated macrophages (TAMs) into M2 type (105). This polarization of M2 macrophages promotes the formation of an immunosuppressive environment and promotes tumor immune escape, Here, miR-155 does this by down-regulating SHIP1 and increasing the secretion of M2-associated cytokines such as IL-10 and TGF-β. These findings establish a relationship between miR-155, HBV infection, and intervention on cytokine release to manage the development of the tumor environment and promotion of HCC.

Enhancing the recruitment of immunosuppressive cells is another mechanism by which ncRNAs contribute to immune evasion and remodeling of the TME. HBx promotes the expression of miR-221 and CXCL12 in HBV-associated HCC and activates the signaling pathway of CXCL12/CXCR4 (18). The latter signaling facilitates the mobilization of the immunosuppressive natural killer T (NKT) cells that enhance the ability of the tumors to evade destruction resulting in growth. By modulating miR-221/CXCL12/CXCR4 signaling, HBx promotes cell growth and immunosuppression in HBV-associated HCC. XIST, a significant oncogenic lncRNA is implicated in HBV-induced HCC and is connected with pyloric ring depth, in addition to the inflammatory TME, particularly CD25+ Tregs (106). Its expression levels were significantly positive with an increase in several immune cells such as CD3, CD4, and CD8 though not significant p value. LncRNA growth activating sequence (GAS) is another lncRNA, which plays a crucial role in immune evasion through activation of immunosuppressive cells for instance Treg cells in TME (107). Lastly, HULC can enhance immune tolerance to the tumor through the activation of PD-1-related immune inhibition. Further, this process is supported by the enhanced secretion of immunosuppressive cytokines, for example, IL-10 and TGF-β1 that promote tumor growth. Thus, targeting the HULC-Treg/PD-1 axis provides a potential strategy to interfere with immune escape to consequently repress HCC progression.

### 3.5 Regulation of tumor stemness

In HBV-related HCC, ncRNAs play key roles in controlling immune escape, stem cell self-renewal, and several signaling pathways that maintain the stem cell-like phenotype of CSCs. Lnc-CCNH-8 plays an important role in this manner by increasing the expression of the checkpoint protein PD-L1 that suppresses the activity of T cells and thereby prevents the destruction of CSCs by the immune system. Lnc-CCNH-8 overexpression sponges miR-217, which negatively regulates PD-L1 synthesis. Thus, the stabilization of PD-L1 in turn promotes immune escape and supports the survival and maintenance of the CSC population. This mechanism is especially important in HBV-induced HCC where the viral protein changes the immune setting to add up further immune evasion and CSC expansion. Under normal conditions, the down-regulation of BMI1, which greatly affects CSCs maintenance, by miR-203a. Nevertheless, miR-203a is down-regulated in HBV-related HCC, particularly in HBsAg-positive tumors. This in turn results in the derepression of BMI1, adding to the self-renewal capabilities of the CSCs. The depletion of miR-203a is also associated with the promotion of stem cell-like phenotype as well as poor prognosis as CSCs become increasingly difficult to treat with chemotherapy and much more efficient at maintaining tumor viability. Likely, SNORD88A & SNORD88B also promotes self-renewal but by a mechanism through nucleolar localization. They contribute towards directing WRN (Werner syndrome ATP-dependent helicase) to the nucleolus to inhibit the Hippo pathway, which is essential for CSC maintenance. Further SNORD88B knockout experiments revealed that the expression of this snoRNA is capable of significantly reducing CSC populations and tumor initiation, implying a stemness-promoting role of this molecule in HCC. Using Lock and Antisense oligonucleotides (ASOs) on SNORD88B has been proven to eliminate CSC-driven tumorigenicity and is hence potential therapeutic option.

In HBV-related HCC, the specific overexpression of MALAT1 is achieved by HBx, and accordingly, the downregulation of miR-124 should exert inhibitory effects on stemness-related pathways. Through down-regulation of miR-124, MALAT1 also promotes activation of the PI3K/Akt signaling pathway that governs self-renewal and apoptosis resistance, key functions of CSCs. This combined interaction between MALAT1 and PI3G/Akt highlights the significance of pathway control in supporting tumor stemness in the HBV-infected cells. Moreover, circRNA cFAM210A acted as a protector of tumor repressor since it suppressed different oncogenic signaling pathways that lead to a stem cell-like state. In normal conditions, cFAM210A binds to and inhibits YBX1, a transcription factor that activates MET signaling involved in stemness. However, in HBV-infected cells, cFAM210A is methylation by HBx and degraded resulting in increased activity of YBX1 and essentially the MET signaling pathway. This dysregulation enables CSCs to proliferate in a given area without any form of regulation and more importantly enhance stemness and tumorigenesis.

## 4 Application of ncRNAs in HBV-related HCC

### 4.1 ncRNAs as diagnostic biomarkers for HCC

**Figure 3:**
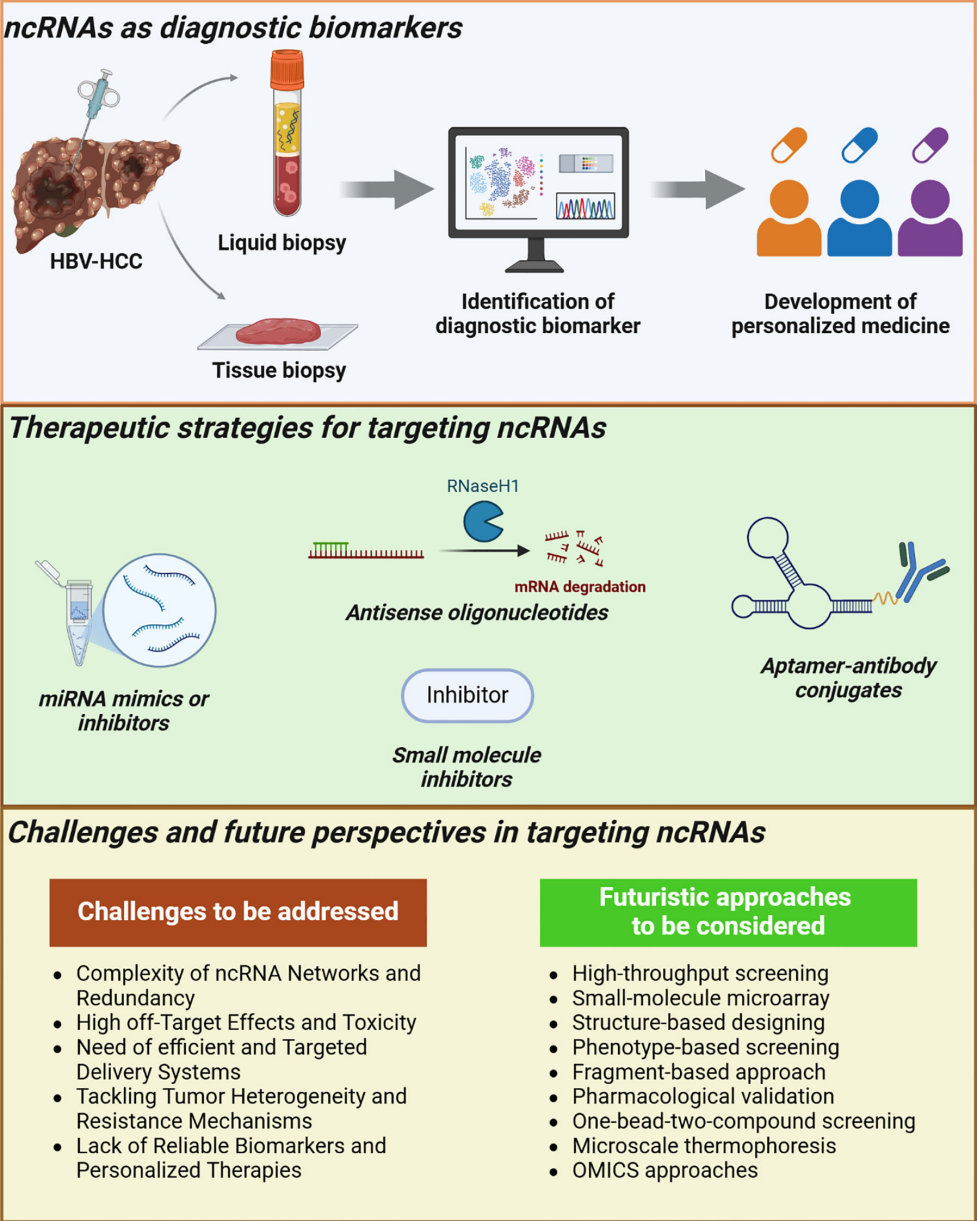
Application of ncRNAs in HBV-related HCC were highlighted. Identification of prognostic biomarkers (lncRNAs, miRNAs, circRNAs) from the HBV-related HCC biopsy samples aid in the development of personalized medicine. Treatment modalities such as the Antisense oligonucleotides (ASO), miRNA mimics and inhibitors, small molecule inhibitors towards ncRNAs can be used to combat the disease progression. Considerable challenges and possible approaches towards ncRNAs therapeutics development were also highlighted.

Generally, in HBV-related HCC patients, the disease condition is mostly diagnosed at very late stages which could have a possible recurrence. In the era of cancer precision medicine, it is very vital to explore significant diagnostic and prognostic biomarkers in the early stages of HBV-related HCC itself for quick decision-making of doctors toward treatment strategies (108, 109). **Table 1** depicts the dysregulated ncRNAs explored as biomarkers of HBV-HCC. Notably, the ncRNAs (miRNAs, lncRNAs, and circRNAs) could potentially function as predictive biomarkers in various tumors, including the HBV-HCC. Tissue samples and blood in circulation of patients are the commonly used clinical samples for the identification of biomarkers, the microarray and qRT-PCR are the widely used detection techniques respectively in HBV-related HCC (110). The area under the curve (AUC) is very important in the development of the biomarkers, while the sensitivity determines the true positive rate (only disease condition) and the specificity correctly identifies a disease-free patient among the test population (111). Notably, a small amount of samples is enough to detect and quantify ncRNAs with high sensitivity and specificity (112). **Figure 3** illustrates the application of ncRNAs in HBV-related HCC.

**Table 1:** Potential dysregulated ncRNAs explored as biomarkers of HBV-HCC.

ncRNA	Name	Sample	Expression pattern	Biomarker	Mechanism	Study type	Reference
miRNA	miR-122-5p	Serum	↓	Prognostic	Regulates apoptosis and metastasis	Computational	(161)
	miR-125b-5p	Serum	↑	Prognostic	Alters tumor growth & proliferation	Computational	
	miR-100-5p	Serum	↑	Prognostic	Regulates apoptosis via targeting PLK1	Computational	
	miR-885-5p	Serum	↑	Prognostic	Modulated angiogenesis via targeting AEG1	Computational	
	miR-148a-3p	Serum	↓	Prognostic	Not clear	Computational	
	miR-101	Serum	↓	Diagnostic	Epigenetic alterations	Preclinical	(162)
	miR-18a	Serum	↑	Prognostic	Targets cell cycle factors	Preclinical	(163)
	miR-93-5p	Urine	↑	Prognostic	Regulates migration via targeting FAT4	Preclinical	(164)
	miR-15a/16	Plasma	↓	Diagnostic	Modulates apoptosis and cellular proliferation	Computational	(165)
	miR-221	Serum	↑	Prognostic	Regulates cell cycle	Patient samples	(166)
	miR-224	Serum	↑	Diagnostic	Alters signaling pathways	Patient samples	(167)
	miR-26a	Tissue	↓	Diagnostic	Regulates inflammatory pathways	Patient samples	(168)
lncRNAs	HEIH	Serum & exosome	↑	Prognostic	Epigenetic alterations	Patient samples	(169)
	HOTAIR	Serum & exosome	↑	Diagnostic	Regulates metastasis & growth	Patient samples	(170)
	LINC00161	Serum & exosome	↑	Diagnostic	Epigenetic alterations and regulates invasion	Patient samples	
	LINC00152	Serum	↑	Diagnostic	Regulates EMT transition	Patient samples	(171)
	AFP	Serum	↑	Diagnostic	Regulates proliferation & cancer stem cell properties	Patient samples	
	UCA1	Serum	↑	Diagnostic	Modulates cell cycle	Patient samples	
	PVT1	Serum	↑	Diagnostic	Modulates cell cycle & apoptosis	Patient samples	(172)
	uc002mbe.2	Serum	↓	Diagnostic	Modulates cell cycle & apoptosis	Patient samples	
	SNHG1	Plasma	↑	Diagnostic	Regulates invasion and proliferation	Patient samples	(172)
circRNAs	hsa_circRNA_104351	Tissue	↑	Diagnostic	Not reported	Patient samples	(173)
	hsa_circRNA_102814						
	hsa_circRNA_103489						
	hsa_circRNA_102109						
	hsa_circRNA_100381						
	hsa_circ_0027089	Plasma	↑	Diagnostic	Regulates cellular proliferation via targeting various miRNAs	Patient samples	(174)
	hsa_circ_0006091	Serum & plasma	↑	Diagnostic	Tumor growth and metabolism	Patient samples	(175)
	circ-ATP5H	Tissue	↑	Therapeutic	Promotes HBV replication via targeting miR-138-5p/TNFAIP3 Axis	Preclinical & Patient samples	(176)
	hsa_circ-000021	Tissue	↓	Prognostic	Modulates metastasis via targeting miR-665/TIMP3 axis	Patient samples	(91)
	circRNA1002	Tissue & serum	↓	Diagnostic	Regulates oxytocin signaling pathway	Computational	(177)

Downregulated (↓); Upregulated (↑).

Recently, Guan et al. reported that the lncRNAs such as ADAMTSL4-AS1, SOCS2-AS1, and AC067931 were strongly overexpressed in HCC tissues and PBMCs, and the HCC is also differentiated from the chronic hepatitis B (CHB) and liver cirrhosis (LC) when combined with alpha-fetoprotein (AFP) confirmed by a qRT-PCR analysis. HCC differentiated from the CHB with an AUC of 0.945, HCC differentiated from the LC with an AUC of 0.871, and HCC differentiated from both CHB and LC with an AUC of 0.905 (113). Likewise, a combined bioinformatics and qRT-PCR analysis of tissues, cells, and whole blood samples of patients with HBV-associated HCC, CHB, and LC revealed that the LINC01793 exerts elevated expression in HBV-associated HCC. The HCC differentiated from the CHB with an AUC of 0.767, and the HCC differentiated from the LC with an AUC of 0.756 respectively (114). Also, the lncRNA HULC, HOTTIP, HOTAIR lnc00152, AFP, lnc00853, lnc00974, lnc00978, lnc01225, DANCR, DGCR5, LncDQ, lnc-GPR89B-15 were potentially reported for their dysregulation and possesses the capacity to act as diagnostic biomarkers of HBV associated HCC (47, 115–117).

Alongside the miRNAs have emerged as significant biomarkers in the detection of HBV-associated HCC. To note, miRNA-21 levels are overexpressed in HCC patient’s serum and plasma samples and significantly differentiated from CHB with an AUC of 0.773, and differentiated from healthy ones with an AUC of 0.953 (118). Two novel non-invasive diagnostic biomarkers miR-122 and miR-224 were also identified in the patient’s plasma at elevated levels. miR-224 in HCC differentiated from the CHB with an AUC of 0.96, and miR-224 in HCC differentiated from the CHB with an AUC of 0.94 (119). In a Japanese cohort study, the miR-21 in plasma samples of patients was significantly overexpressed and differentiated the HCC from CHB with an AUC of 0.773 and differentiated the HCC from healthy ones with an AUC of 0.953 (118). While, in the Egyptian cohort study, miR-21 in the serum samples from patients was significantly overexpressed and differentiated the HCC from CHB with an AUC of 0.943. These studies indicate the significance of exploring miR-21 as the diagnostic biomarker of HBV-related HCC (119). Also, the miRNAs miR-9-3p, miR-29a, miR-34a, miR-92a-3p, miR-424, miR-665, and miR-3126-5p were potentially reported for their dysregulation and hold promise as diagnostic biomarkers of HBV-related HCC (120–122).

Recently, due to their significant properties such as non-invasive, highly stable, significant dysregulation in disease, circulation, and tissue-specific expression nature, the circRNAs are widely recognized as potential diagnostic biomarkers in HBV-associated HCC. circRNAs can be quantified in plasma, serum, and plasma exosomes of the patients. In a study, it was reported that the circ_0004277 in the plasma exosome of the patient was significantly upregulated and differentiated the HCC from healthy ones with an AUC of 0.816 (123). However, the circ_0028861 down expression was observed in the serum exosome of HCC patients than in the HBV and LC conditions. The HCC was differentiated from CHB with an AUC of 0.83, the HCC was differentiated from LC with an AUC of 0.75, and the HCC was differentiated from both CHB and LC with an AUC of 0.79 (124). In another Chinese cohort study, researchers reported higher expression of circ_0070396 in the plasma exosome of HCC patients than in the HBV and LC conditions. Here, the HCC was differentiated from CHB with an AUC of 0.774, the HCC was differentiated from LC with an AUC of 0.661, and the HCC was differentiated from healthy ones with an AUC of 0.857 (125). Also, the circRNAs circ_0051443, circ_0072088, circ_104075, circANTXR1, circSMARCA5, circPTGR1, circWHSC1, circ_0004001, circ_0004123 and circ_0075792 were potentially reported for their dysregulation and holds promise as diagnostic biomarkers of HBV-related HCC (126–129).

### 4.2 Therapeutic strategies targeting ncRNAs

Therapeutic modalities such as the Antisense oligonucleotides (ASO), miRNA mimics and inhibitors, and small molecule inhibitors towards lncRNAs, miRNAs, and circRNAs have emerged as promising strategies due to their therapeutic potential (130, 131). ASO are sequence-specific therapeutics that are designed from the ssDNA and target the mRNA target and activate TLR via CpG motif, miRNA therapeutics are RISC complexed associated ans that are designed from the ssRNA to promote TLR7/8 stimulation, while the inhibitors directly bind to the lncRNAs and circRNAs and inhibits their activity in various cancers including the HBV-related HCC (130).

The hsa_circ_0000517 sponges with miR-326, regulate the production of SMAD6 in HBV-related HCC. Elevated hsa_circ_0000517 expression was associated with reduced miR-326 levels, while SMAD6 was found to be triggered in HCC. In contrast, the downregulation of hsa_circ_0000517 hinders cell proliferation, invasion, and migration. Notably, the hsa_circ_0000517 mediated regulations of SMAD6 were shown to counteract the suppressive effect of miR-326 mimics (132). A liposomal miR-34a mimic (MRX34), currently in a Phase I trial, has reported data on the maximum tolerated dose, safety profile, and pharmacokinetics of miR-34a in HBV-associated HCC. The miR-326 mimics combination with dexamethasone showed acceptable safety and efficacy, and the maximum tolerated dose was estimated to be 93 mg/m^2^ in patients (133). In HCC cells and xenograft animal models, the miR-361-5p was observed to be downregulated and modulate the production of Chemokine (C-X-C Motif) receptor 6 (CXCR6). These miR-361-5p mimics were transferred through lentivirus transfection, and the stimulated miR-361-5p overexpression suppressed the HCC progression (134). miR-375 is generally down-expressed in HBV-HCC, and it is forced expressed to inhibit the tumor growth by promoting apoptosis and inhibiting metastasis respectively. Cholesterol-conjugated 2’O-methyl modified miR-375 mimics through lentiviral transfection into HCC cells and patient tissue samples demonstrated that it targets and regulates the astrocyte elevated gene-1 (AEG-1) expression levels (135).

miR-422a mimics transferred through lentivirus transfection were conducted to explore the impact of miR-422a in HBV-related HCC. miR-422a is reduced in HCC and targets forkhead box G1 (FOXG1), FOXQ1, and FOXE1 and its upregulation inhibits the metastasis and tumor progression. Also, they reported that the miR-422a expression is regulated by FOXG1, FOXQ1, and FOXE1 and claims an established double-negative feedback loop (136). While the miR-98-5p mimics overexpression inhibited proliferation, and metastasis and promoted apoptosis in MHCC97H-HBV cells. Also, it reduces the HBV DNA levels and significantly inhibits tumor growth in xenograft mice models (137). Alongside some miRNA inhibitors are also being developed and studied in preclinical models. A very interesting study used a combination of miR-122 mimic/miR-221 against HCC, which inhibited proliferation, and angiogenesis and reduced the production of cell cycle modulators like cyclin D1 and TGF-β respectively. This combination potentially reduces tumor progression by targeting centrin-specific protease 1 (SENP1) and ADP-ribosylation factor 4 (ARF4) and decreasing the production of α-smooth muscle actin and tropomyosin-1 confirmed in both laboratory and animal model studies (138).

Sorafenib, Cabozantinib, Lenvatinib, and Regorafenib are some commonly available drugs for HBV-associated HCC that target various targets including the vascular endothelial growth factor (VEGF), platelet-derived growth factor receptors (PDGFRs), epidermal growth factor receptor (EGFR) and fibroblast growth factor receptor (FGFR) respectively (139). Current research focuses on the development of potent inhibitors of lncRNAs and circRNAs to precisely develop targeted cancer medicine. The lncRNAs and circRNAs show significant binding potential towards their mRNA targets and regulate their expression in various cancers including HBV-related HCC (140). Small molecule inhibitors (less than 500 M.W) demonstrated significant potential to specifically bind towards the target lncRNAs and circRNAs and inhibit their binding towards mRNA targets, thus reducing the activity of oncogenic ncRNAs.

Current and historical data from a few clinical trials highlight the translational potential of ncRNA-based therapies for HCC. A good example is the Phase I clinical trial of MRX34 (liposomal miR-34a mimic), which has shown an anti-tumor effect in advanced solid tumors such as HCC (NCT01829971). However, the trial was stopped rather prematurely due to immune-related adverse events of the severe grade, which indicates the problem of implementation of RNA-based therapies into clinical practice.

It is notwithstanding these breakthroughs that clinical translation is still hindered by a number of barriers. (1) lack of stable and specific delivery vehicles that will selectively target liver tumors without off-target accumulation; (2) heterogeneous microenvironments in tumors, particularly in HBV-HCC that restrict the reproducibility of response; and (3) complex regulatory context for the long-term safety efforts regarding RNA therapeutics. Further, the patient stratification availability utilizing ncRNA expression levels is yet at the infant stage, thus restricting the application of personalized treatments. To overcome these barriers future efforts should concentrate on the incorporation of precision delivery systems, real-time monitoring of ncRNA biomarkers, and therapy combination with immune checkpoint inhibitors or kinase inhibitors. Further, designing multi-center, ethnically diverse clinical trials will be essential to the broader applicability of ncRNA-targeted therapeutics in HBV-HCC at the global level.

### 4.3 Challenges and future perspectives in targeting ncRNAs for HCC therapy

The therapeutic targets of ncRNAs (lncRNAs, miRNAs, and circRNAs) have become a potential approach, with several investigations ongoing to evaluate some important concerns such as safety, efficacy, delivery, specificity, sensitivity, and tolerability among patients (130). Below some of the challenges for the therapeutic targeting of ncRNAs in HBV-related HCC have been highlighted (141–145). First, the complexity of ncRNA networks makes making the single ncRNA targeted therapy challenging, and the redundancy may lead to off-target effects (146). Both lncRNAs and circRNAs can act as molecular sponges for miRNAs, complicating the prediction of downstream pathway effects and raising the potential for off-target events. For instance, miRNA mimics or inhibitors, due to partial sequence complementarity, may inadvertently silence unintended mRNA targets, potentially triggering toxic responses. A case in point is the clinical trial of MRX34, a liposomal mimic of miR-34a, which was prematurely halted due to severe immune-related adverse events (NCT01829971), despite promising preclinical results in liver cancer models. Second, is the off-target effects and toxicity of miRNA mimics or miRNA inhibitors, which can bind with several mRNA target sequences due to their partial sequence complementarity promoting the silencing of unwanted mRNAs. Also, the delivery of these ncRNAs is often associated with adverse immune responses. Notably, the miR-34a mimics showed potent activity against HCC, however, it was halted in clinical trials due to immune-related adverse effects (133). Third, is the development of efficient and targeted delivery systems of ncRNAs to maintain their stability and degradation. Present approaches such as exosome-based delivery, aptamer-conjugated nanoparticles, and viral vectors have to be further optimized to reduce toxicity and increase efficacy. Fourth, is the tackling of tumor heterogeneity and resistance mechanisms of the HBV-HCC. For instance, single ncRNA inhibition may possess reduced tumor growth at initial stages and has a strong chance of bypassing the ncRNA/mRNA axis leading to therapeutic resistance. Fifth, is the absence of trustworthy biomarkers and personalized therapies, indicating the importance of the development of diagnostic and prognostic biomarkers of HBV-related HCC to identify the disease in its early stages itself. Extensive ncRNA profiling among various cohorts could help us in the development of personalized treatment strategies. By addressing these challenges, we could able to develop more precise treatments for HBV-associated HCC.

Unlike the protein’s structural complexity, the ncRNAs have a simple structure that provides enough space for the small molecule inhibitors to bind and modulate the function of ncRNAs. Targeting the ncRNAs using small molecule inhibitors has emerged as a potential strategy, but still lacks more evidence to develop as a treatment modality (147). Various methodologies, such as high-throughput screening (HTS), small-molecule microarray (SMM) techniques, structure-based design, phenotype-based investigation, fragment-based strategies, pharmacological validation, one-bead-two-compound (OBTC) screening, microscale thermophoresis, and OMICS-based approaches, can be employed to develop effective inhibitors targeting ncRNAs (148). Recently, researchers developed a deep learning algorithm named DeepdlncUD, for the the precise prediction of mechanisms by which small molecule inhibitors modulate lncRNA expression. This computational tool will aid in the development of potent lncRNA inhibitors with its 9 deep-learning algorithms (149). More computational tools have to be developed in the future to guide the design and advancement of innovative therapeutic interventions targeting ncRNAs for the precise management of HBV-related HCC.

Other than various experimental procedures, computational methods have also proved useful in the identification of biomarkers in HBV-associated HCC. Integrated bioinformatics pipelines based on the transcriptomic, epigenomic, and proteomic data have helped find the diagnostic and prognostic ncRNA signatures. For instance, there was a recently published integrative transcriptomic and proteomic research that identified the novel miRNA and lncRNA signatures to estimate tumor grade and overall survival in HCC patients (150). The other research used both combined epigenomic and transcriptomic profiling to identify methylation-regulated lncRNAs underlying HBV-mediated oncogenesis (151). Such improvements emphasize the importance of OMICS in the identification of ncRNA biomarkers for designing personalized treatment in HBV-HCC.

Furthermore, some of these machine learning models used to classify HBV-HCC from chronic hepatitis B or cirrhosis include SVMs, random forests, and LASSO regression which when utilized have yielded success (152). For example, ensemble models have been trained on public datasets to find miR-21, miR-122, and LINC00152 to be key discriminators with high diagnostic accuracy (AUC> 0.90) (153). In the recent past, various deep learning frameworks like DeepHCC have been developed to predict HCC-specific ncRNA biomarkers via the use of convolutional neural networks (CNNs) trained on high-throughput sequencing data (154, 155). Such tools allow for the prioritization of candidate biomarkers with minimal manual curation, increase reproducibility, and help design precision medicine strategies. In the future, the integration of computational modeling and large, diverse patient cohorts will be key to fine-tuning and validating ncRNA-based panels for clinical application.

In overcoming delivery and specificity problems, new-developing technologies like CRISPR-based ncRNA and exosome-based delivery systems have shown promising prospects. CRISPR-Cas13d and CRISPR-CasRx may be engineered to deter destructive ncRNAs with a great degree of accuracy, as a result of which off-target effects can be reduced and programmed obstructive of cancerous transcripts can be delivered (156). These tools have already demonstrated efficacy in cancer models in selective disruption of these lncRNAs and circRNAs responsible for facilitating tumor growth (157, 158). Additionally, the development of catalytically inactive dCas proteins that are fused with RNA modulatory domains allows epigenetic control of ncRNA expression without causing double-strand breaks.

Simultaneously, the exosome-based delivery systems serve as a biocompatible and targeting tool for ncRNA mimics or inhibitors. It has been demonstrated that engineered exosomes can effectively infiltrate the tumor microenvironment, escape from immune detection, and deliver the therapeutic cargo at very low levels of toxicity; such exosomes could be loaded with miRNAs or siRNAs. For example, the exosomal delivery of miR-122 and miR-199a has shown efficacy in hepatoma models amounting to reversing drug resistance and sensitization of tumor to sorafenib (159, 160). Such advanced platforms in conjunction with artificial intelligence and high throughput screening may deliver a new age of precision RNA therapy in HBV-HCC. Such technologies need further development and clinical validation if there are to be bridges between experiential promise and clinical reality.

Despite the rising potentials of ncRNAs in HBV-HCC diagnostics and therapeutics are faced with multifarious challenges discouraging their clinical translation. Technically the sensitive detection of ncRNAs, especially in liquid biopsies continues to be challenging due to their low quantity, instability, and lack of standardized protocol on platforms used like qRT-PCR, microarrays, and next-gen sequencing. The separation of ncRNAs produced by tumors from those produced by stromal or immune cells makes biomarker interpretation even more confusing. Biologically, redundancy, as well as pleiotropy, represent serious bottlenecks; there are many ncRNAs with overlapping functions, that target the same mRNAs or signaling pathways, so the attribution of effects to a specific molecule is challenging. Moreover, although tissue-specific expression of ncRNAs can provide a level of biomarker accuracy, it also complicates the development of systemically delivered approaches because of nonspecific effects in non-tumor tissues. Clinically, patient heterogeneity will inhibit the generalizability of ncRNA-based interventions. The lack of strong patient stratification methods involving ncRNA signatures disables personalized treatment strategies. Moreover, many of the existing therapeutic approaches of concentration are single-agent ncRNA directed, which may be inefficient in addressing the complex and adaptive nature of HBV-HCC. New prospects should focus on integrative strategies where the ncRNA therapeutics are coupled with the immune checkpoint inhibitors or the kinase inhibitors, together with progressive delivery systems and multi-omics-directed patient classifications to overcome such translational barriers. Overall, **Table 2** enlists the ncRNAs-mediated regulatory networks in HBV-HCC.

**Table 2:** ncRNAs-mediated regulatory networks in HBV-HCC.

ncRNA	Target	Pathway	Mechanistic function	Reference
miR-1	HDAC4, E2F	Cell cycle regulation	Induces cell cycle arrest and regulates proliferation	(178)
miR-21	PDCD4, PTEN	MAPK, PI3K	Promotes tumorigenesis by auto-regulatory mechanism	(179)
miR-29	Bcl-2 and Mcl-1	Intrinsic mitochondrial pathway	Promotes apoptosis	(180)
miR-101	Mcl-1	MAPK	Promotes apoptosis and suppresses tumorigenicity	(181)
miR-122	Cyclin G1	Cell cycle regulation	Suppresses tumor cell growth	(182)
miR-125	Mcl-1, IL6R	IL6R-mediated signal pathway	Induces apoptosis and inhibit proliferation	(183)
miR-152	DNMT1	MAPK	Epigenetic aberrations	(184)
miR‐155‐5p	PTEN	PI3K/Akt	Regulates invasion & metastasis	(185)
	SOCS1	JAK/STAT	Regulates tumor growth & proliferation	(186)
miR-192	SLC39A6	SNAIL	Suppresses metastasis & growth	(187)
	ZEB2	p53 pathway	Regulates EMT transition	(188)
miR-199a-3p	mTOR, c-Met	mTOR	Suppresses tumor growth & invasion; induces cell cycle arrest	(189)
miR-221	ERα	Estrogen signaling pathway	Promotes proliferation & tumor growth	(190)
miR-372/373	NFIB	NFIB -mediated signal pathway	Regulates tumor growth & proliferation	(191)
let-7	STAT3	JAK/STAT	Regulates proliferation & tumorigenesis	(192)
XIST	miR-192→ TRIM25	TGF-β & p53	Regulates tumor growth & proliferation	(193)
LIF	miR-192-5p→ CYR61	PI3K/Akt	Regulates apoptosis & tumor microenvironment	(194)
HULC	miR-372→ PPARA	Lipid metabolism	Promotes angiogenesis & inhibits tumor growth	(195)
MALAT1	miR-200a→ FOXA2/CDK6	Wnt/β-catenin	Regulates EMT transition & metastasis	(196)
HEIH	miR-129→ EZH2	TGF-β & p53	Regulates apoptosis & metastasis	(197)
circ_0067934	miR1324→FZD5	Wnt/β-catenin	Regulates tumor growth & metastasis	(198)
circ_0008934	miR-1305→TMTC3	GRP78/PERK	Regulates tumor growth & metastasis	(199)
circ_0005397	miR-326→PDK2	PI3K/Akt	Regulates apoptosis, invasion and migration	(200)
circ_0001073	mir-511-5p→ LIFR	PI3K-Akt	Promotes growth & metastasis	(201)
SNORD126	FGFR2	PI3K-AKT	Regulates tumor growth & apoptosis	(202)
SNORD113-1	ERK1/2, SMAD2/3	MAPK & TGF-β	Regulates apoptosis & tumorigenesis	(203)
SNORD52	CDK1	Sucrose metabolism	Induces cell cycle arrest and regulates proliferation	(204)

## 5 Conclusion

The intricate functions of ncRNAs in the advancement of HBV-related HCC underscore their critical importance as both drivers of tumorigenesis and potential therapeutic targets. By modulating processes like cell proliferation, apoptosis suppression, tumor spread, immune evasion, and stemness maintenance, ncRNAs contribute significantly to the aggressive nature of HBV-associated HCC. The unique regulatory mechanisms of miRNAs, lncRNAs, circRNAs, and snoRNAs provide a comprehensive framework to understand HBV-related HCC pathogenesis, offering valuable insights for diagnostic and therapeutic innovations. The current body of evidence underscores their promise not only as mechanistic mediators but also as clinically actionable biomarkers and therapeutic targets. The recent rise in clinical studies exploring circulating miRNAs, lncRNAs, and circRNAs as non-invasive biomarkers for early detection and prognosis has enhanced the clinical potential of ncRNA-based liquid biopsies.

Despite advancements in delineating the roles of ncRNAs, several challenges and opportunities remain. A more comprehensive understanding of the context-specific roles of ncRNAs, particularly in patient-derived models, is essential to overcome variability in their expression and function. Furthermore, the integration of ncRNA-based biomarkers into clinical practice necessitates robust validation studies to establish their reliability, sensitivity, and specificity.

From a therapeutic standpoint, novel approaches such as antisense oligonucleotides, RNA mimics, and small molecule inhibitors targeting ncRNAs hold promise. However, difficulties like delivery efficiency, off-target effects, and immune activation should be addressed to enhance their clinical viability. The development of combination therapies that target ncRNA regulatory networks alongside existing antiviral and anticancer treatments may further improve outcomes for patients with HBV-related HCC.

Looking ahead, future research should prioritize the integration of multi-omics datasets with AI-based predictive algorithms to identify robust and personalized ncRNA signatures for HBV-HCC. Moreover, the combination of ncRNA-targeted therapies with existing immunotherapies and kinase inhibitors may overcome resistance and improve efficacy. Expanding clinical trials across multi-ethnic cohorts will also ensure global applicability and reduce population bias in biomarker development.
