# Correction: Non-coding RNAs as key regulators in hepatitis B virus-related hepatocellular carcinoma

**DOI:** 10.3389/fimmu.2025.1653985

**Published:** 2025-07-08

**Authors:** Prasanna Srinivasan Ramalingam, Liming Zhang, Md Sadique Hussain, Gyas Khan, Wedad Mawkili, Ali Hanbashi, Gaurav Gupta, Purushothaman Balakrishnan, Sivakumar Arumugam

**Affiliations:** ^1^ Protein Engineering Lab, School of Biosciences and Technology, Vellore Institute of Technology, Vellore, Tamil Nadu, India; ^2^ School of Basic Medical Sciences, Tsinghua University, Beijing, China; ^3^ Uttaranchal Institute of Pharmaceutical Sciences, Uttaranchal University, Dehradun, Uttarakhand, India; ^4^ School of Pharmaceutical Sciences, Lovely Professional University, Phagwara, Punjab, India; ^5^ Department of Pharmacology and Toxicology, College of Pharmacy, Jazan University, Jazan, Saudi Arabia; ^6^ Centre for Research Impact and Outcome-Chitkara College of Pharmacy, Chitkara University, Rajpura, Punjab, India; ^7^ Centre of Medical and Bio-allied Health Sciences Research, Ajman University, Ajman, United Arab Emirates; ^8^ Department of Biomaterials, Saveetha Dental College and Hospitals, SIMATS, Saveetha University, Chennai, India

**Keywords:** biomarkers, hepatitis B virus, hepatocellular carcinoma, non-coding RNAs, therapeutic targets

In the published article, there were several errors in the affiliations. Affiliation “School of Basic Medical Sciences, Tsinghua University, Beijing, China” was omitted for author “Liming Zhang”. This affiliation has now been added for author “Liming Zhang”.

The affiliation “School of Pharmaceutical Sciences, Lovely Professional University, Phagwara, Punjab, India” was also omitted for author “Md Sadique Hussain”. This second affiliation has now been added for author Md Sadique Hussain.

Additionally, author “Liming Zhang” was erroneously assigned to affiliation “School of Pharmaceutical Sciences, Lovely Professional University, Phagwara, Punjab, India”. This affiliation has now been removed for author “Liming Zhang”.

The authors apologize for this error and state that this does not change the scientific conclusions of the article in any way. The original version of this article has been updated.

